# Short-term plasticity of visuo-haptic object recognition

**DOI:** 10.3389/fpsyg.2014.00274

**Published:** 2014-04-02

**Authors:** Tanja Kassuba, Corinna Klinge, Cordula Hölig, Brigitte Röder, Hartwig R. Siebner

**Affiliations:** ^1^Danish Research Centre for Magnetic Resonance, Copenhagen University Hospital HvidovreHvidovre, Denmark; ^2^NeuroImageNord/Department of Systems Neuroscience, University Medical Center Hamburg-EppendorfHamburg, Germany; ^3^Department of Neurology, Christian-Albrechts-UniversityKiel, Germany; ^4^Department of Psychiatry, Warneford HospitalOxford, UK; ^5^Biological Psychology and Neuropsychology, University of HamburgHamburg, Germany

**Keywords:** multisensory interactions, visual perception, haptic perception, object recognition, repetitive transcranial magnetic stimulation, functional magnetic resonance imaging

## Abstract

Functional magnetic resonance imaging (fMRI) studies have provided ample evidence for the involvement of the lateral occipital cortex (LO), fusiform gyrus (FG), and intraparietal sulcus (IPS) in visuo-haptic object integration. Here we applied 30 min of sham (non-effective) or real offline 1 Hz repetitive transcranial magnetic stimulation (rTMS) to perturb neural processing in left LO immediately before subjects performed a visuo-haptic delayed-match-to-sample task during fMRI. In this task, subjects had to match sample (S1) and target (S2) objects presented sequentially within or across vision and/or haptics in both directions (visual-haptic or haptic-visual) and decide whether or not S1 and S2 were the same objects. Real rTMS transiently decreased activity at the site of stimulation and remote regions such as the right LO and bilateral FG during haptic S1 processing. Without affecting behavior, the same stimulation gave rise to relative increases in activation during S2 processing in the right LO, left FG, bilateral IPS, and other regions previously associated with object recognition. Critically, the modality of S2 determined which regions were recruited after rTMS. Relative to sham rTMS, real rTMS induced increased activations during crossmodal congruent matching in the left FG for haptic S2 and the temporal pole for visual S2. In addition, we found stronger activations for incongruent than congruent matching in the right anterior parahippocampus and middle frontal gyrus for crossmodal matching of haptic S2 and in the left FG and bilateral IPS for unimodal matching of visual S2, only after real but not sham rTMS. The results imply that a focal perturbation of the left LO triggers modality-specific interactions between the stimulated left LO and other key regions of object processing possibly to maintain unimpaired object recognition. This suggests that visual and haptic processing engage partially distinct brain networks during visuo-haptic object matching.

## Introduction

An object's geometrical structure (shape) and surface can be extracted by both using vision and haptics. Integrating shape information across senses can facilitate object recognition (Stein and Stanford, 2008). In vision, the lateral occipital complex (LOC), consisting of subregions in the lateral occipital cortex (LO) and in the fusiform gyrus (FG) (Malach et al., 2002), has long been known to show a preferential response to images of objects as opposed to their scrambled counterparts or other textures (Malach et al., 1995; Grill-Spector et al., 1999; Kourtzi and Kanwisher, 2001). Subsequent neuroimaging studies and studies using transcranial magnetic stimulation (TMS) have linked object- or shape-specific brain responses in the LOC to individual performance during visual object recognition (Grill-Spector et al., 2000; Bar et al., 2001; Ellison and Cowey, 2006; Williams et al., 2007; Pitcher et al., 2009). The functional relevance of the LOC has been further substantiated by patients with lesions in the occipito-temporal cortex suffering from visual agnosia, that is, a severe deficit in visually recognizing objects despite otherwise intact intelligence (Goodale et al., 1991; Karnath et al., 2009). Object-specific responses in the LOC, particularly in the left LO, have also been found when comparing brain responses during the haptic exploration of objects and texture stimuli (Amedi et al., 2001, 2002; Kassuba et al., 2011) or when testing for haptic shape adaptation (Snow et al., 2013). Accordingly, lesions in occipito-temporal cortex can lead to haptic object agnosia (Morin et al., 1984; Feinberg et al., 1986) but see (Snow et al., 2012). Since both vision and haptics provide shape information, it has been proposed that the left LO comprises multisensory representations of object shape that are accessed by the different senses (Amedi et al., 2002). Accordingly, the left LO is typically not recruited by auditory object stimuli which do not provide any shape information unless subjects have learned to extract shape information from soundscapes produced by visual-to-auditory sensory substitution devices (Amedi et al., 2002, 2007).

Previous studies had neglected potential intrinsic differences in the relative contributions of vision and haptics to visuo-haptic shape or object recognition. Since vision provides information about several object features in parallel and even if the object is outside the reaching space, there might be an overall dominance of vision in object recognition, at least if objects have to be recognized predominantly based on their shape. In line with this notion, we have recently found an asymmetry in the processing of crossmodal information during visual and haptic object recognition (Kassuba et al., 2013a). Using a visuo-haptic delayed-match-to-sample task during functional magnetic resonance imaging (fMRI), the direction of delayed matching (visual-haptic vs. haptic-visual) influenced the activation profiles in bilateral LO, FG, anterior (aIPS) and posterior intraparietal sulcus (pIPS), that is, in regions which have previously been associated with visuo-haptic object integration (Grefkes et al., 2002; Saito et al., 2003; Stilla and Sathian, 2008; Kassuba et al., 2011; for review see Lacey and Sathian, 2011). Only when a haptic target was matched to a previously presented visual sample but not in the reverse order (i.e., when a visual target was matched to a haptic sample) we found activation profiles in these regions suggesting multisensory interactions. In line with the maximum likelihood account of multisensory integration (Ernst and Banks, 2002; Helbig and Ernst, 2007), we attributed this asymmetry to the fact that haptic exploration is less efficient than vision when recognizing objects based on their shape (given highly reliable input from both modalities) and gains more from integrating additional crossmodal information than vision.

To further explore the role of left LO in visuo-haptic object integration, we here examined how repetitive TMS (rTMS) of the left LO affects crossmodal object matching. Specifically, we applied real or sham (non-effective) offline 1 Hz rTMS to the left LO immediately before subjects performed a visuo-haptic delayed-match-to-sample task during fMRI. The published results reported above (Kassuba et al., 2013a) present the results after sham rTMS, the current paper focuses on how these multisensory interaction effects were modulated by real rTMS. During fMRI, a visual or haptic sample object (S1) and a visual or haptic target object (S2) were presented sequentially, and subjects had to indicate whether the identity of both objects was the same (congruent) or not (incongruent). Thus, the event of matching (processing S2 and matching it to previously presented S1) was manipulated by three orthogonal factors: (1) S1 and S2 were from the same (unimodal) or different modalities (crossmodal), (2) their identity was congruent or incongruent, and (3) S2 was presented either in the visual or the haptic modality. We assumed that crossmodal integration occurs only when the visual and haptic object inputs are semantically congruent (Laurienti et al., 2004). Multisensory interactions were defined as an increased activation during crossmodal vs. unimodal matching (crossmodal matching effects, cf. Grefkes et al., 2002) that were stronger for congruent than incongruent object pairs (crossmodal matching by semantic congruency interaction; Kassuba et al., 2013a,b). The rTMS-induced changes in task-related activity were investigated with blood-oxygenated-level-dependent (BOLD) fMRI. We hypothesized that real compared to sham rTMS of the left LO would trigger compensatory increases in activity not only at the site of stimulation but additionally in remote key regions of visuo-haptic object integration such as the right LO, bilateral FG, and IPS. Based on previous work (Kassuba et al., 2013a), we predicted that real rTMS would particularly affect visuo-haptic interactions during matching of haptic as opposed to visual S2.

## Materials and methods

### Subjects

The description of the subjects is reproduced from (Kassuba et al., 2013a: Participants, p. 60) and adjusted to include rTMS-specific information. Nineteen healthy right-handed volunteers took part in this study. In one female subject, real rTMS caused uncomfortable sensations on her skull and the experiment was aborted. Data acquisition was successfully completed in 18 participants (9 females, 22–33 years of age, average 25.72 ± 2.87). All subjects reported normal or corrected-to-normal vision, normal tactile and hearing ability, and none had a history of psychiatric or neurological disorders. Handedness was assessed with the short form of the Edinburgh Handedness Inventory (Oldfield, 1971). All subjects were right-handed [Laterality Index ≥ 0.78; scaling adapted from Annett (1970)]. Written informed consent was obtained from each subject prior to the experiment. The study protocol was approved by the local ethics committee (Ärztekammer Hamburg).

### Experimental procedures

**Figure 1:**
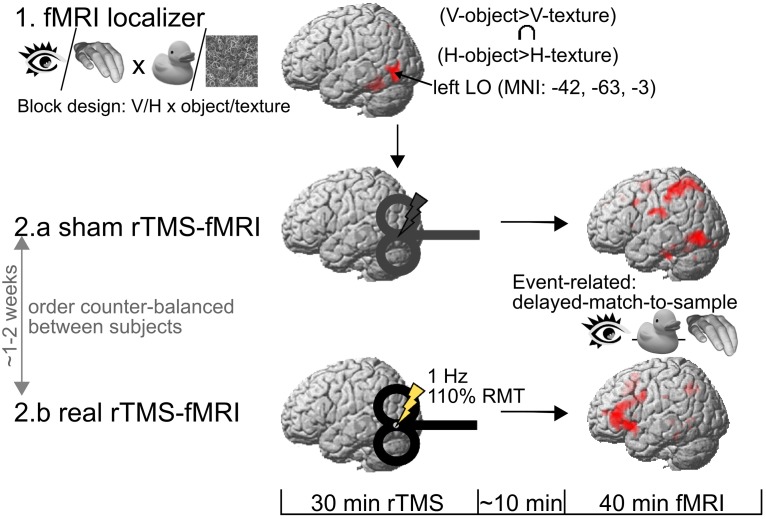
**Experimental procedure**. One day after a behavioral training session (not illustrated here), subjects took part in an fMRI localizer in order to identify the rTMS target area in the left lateral occipital cortex (LO). The rTMS target area was defined by the convergence of visual (V) and haptic (H) processing of objects vs. textures in the left LO. Then, the subjects underwent two 40 min event-related fMRI sessions on separate days (at least one week apart), both preceded by an off-line session of either real or sham 1 Hz rTMS to the left LO. RMT, motor resting threshold.

The description of the experimental procedures is reproduced from Kassuba et al. (2013a: Experimental design and procedure, p. 60) with slight changes in phrasing. All subjects took part in four experimental sessions which were conducted on separate days (Figure 1). First, subjects attended a behavioral training session. An epoch-related fMRI localizer session was performed a day later. The last two sessions each consisted of a 40 min run of event-related fMRI, preceded by either 30 min of sham or real rTMS. The order of the real and sham rTMS sessions was counterbalanced across subjects and separated by at least one week.

In the initial training session, subjects were trained outside the MRI scanner room to recognize 24 object stimuli by viewing photographs and by haptic exploration with an appropriate speed (without ever viewing the real objects themselves). The training was repeated until the object stimuli were identified with an accuracy of 100% (0-1 repetitions per subject and object). In addition, subjects were familiarized with the visual and haptic texture stimuli used in the localizer fMRI session to be presented on the next day. We ran this training to avoid confounding effects due to differences in familiarity and recognition performance between the two modalities.

### Epoch-related fMRI localizer

The description of the localizer is reproduced from (Kassuba et al., 2013a: Visuo-haptic fMRI localizer, p. 60) with slight changes in phrasing. The left LO (rTMS target region) and further regions of interest (ROIs) were identified by means of an fMRI localizer. The paradigm determined the convergence of brain activation during unimodal processing of visual and haptic object stimuli as compared to non-object control stimuli of the same modality. In different blocks, we presented visual, haptic or auditory object or corresponding texture stimuli, resulting in six different block conditions: visual-object, haptic-object, auditory-object, visual-texture, haptic-texture, and auditory-texture. Within each block condition, subjects had to press a button whenever an identical stimulus was presented in two consecutive trials (1-back task, responses in 12.5% of trials). Each stimulation block lasted 30 s during which 8 stimuli from the respective condition were presented (2 s stimuli + 2 s inter-stimulus-interval). The subjects were informed 2.8 s before each block by a visual instruction (0.8 s) about the upcoming block and whether they would see (picture of an eye), touch (picture of a hand) or hear (picture of an ear) stimuli. Each stimulation block was followed by 11.5 s of rest, and each blocked condition was presented six times. The left LO was destined as the peak of the group mean BOLD response in the conjunction contrast (visual-object > visual-texture) ∩ (haptic-object > haptic-texture) at *p* < 0.001, uncorrected (MNI coordinates in mm: *x* = −42, *y* = −63, *z* = −3). The auditory stimuli were used in the context of a different research question (these results have been previously published in Kassuba et al., 2011).

### Event-related fMRI experiment

The description of the event-related fMRI experiment is reproduced from (Kassuba et al., 2013a: Event-related fMRI experiment, pp. 60–62) with slight changes in phrasing. The main fMRI experiment entailed two experimental sessions that used an identical event-related fMRI paradigm (except for differences due to pseudorandomization of the conditions and stimuli). Each experiment started with a short practice session, consisting of a short recall of the initial training, and then subjects were familiarized with the subsequent fMRI task. Thereafter, real or sham 1 Hz rTMS was applied to the left LO for 30 min followed by the event-related fMRI experiment (for details on rTMS see Repetitive TMS).

**Figure 2:**
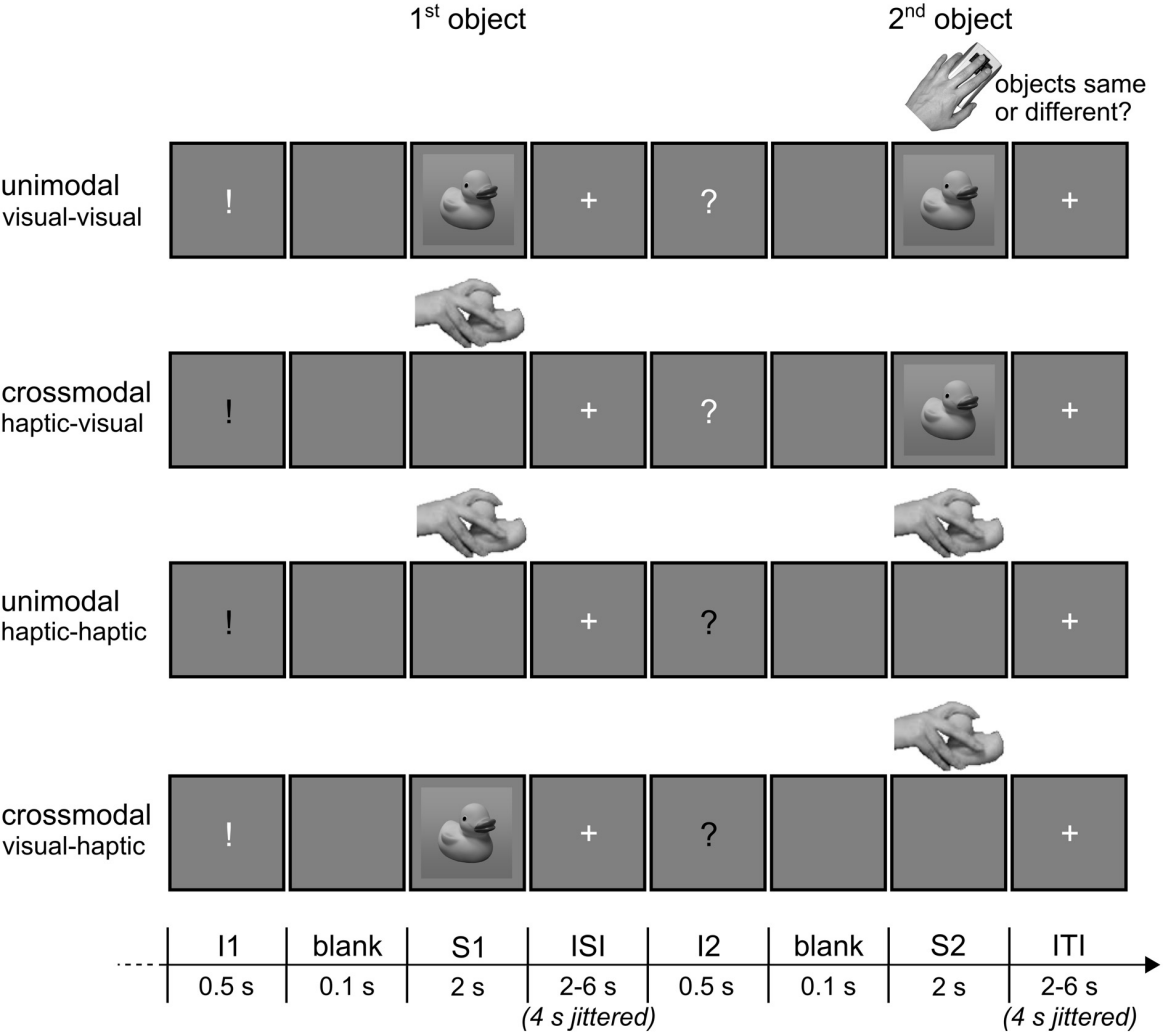
**Illustration of the event-related fMRI paradigm**. Each trial consisted of a sample (S1) and a target object stimulus (S2), and subjects had to decide by button press whether the two objects were congruent (50%) or incongruent (50%). S1 and S2 were either haptic or visual stimuli, and both could be presented either within the same modality (unimodal) or across modalities (crossmodal). A white or black visually presented exclamation (I1) and question mark (I2) before the stimuli informed the subjects about the sensory modality S1 and S2, respectively, would be presented in. ISI, inter-stimulus-interval; ITI, inter-trial-interval. Reprinted from NeuroImage, 65, Kassuba et al., Vision holds a greater share in visuo-haptic object recognition than touch, p. 61, Copyright Elsevier Inc. (2013a), with permission from Elsevier.

Example trials of the event-related fMRI paradigm are shown in Figure 2. Each trial consisted of a sample object stimulus (S1) and a target object stimulus (S2) presented successively, and the subjects' task was to decide whether or not both stimuli referred to the same object (50% congruent and 50% incongruent). The object stimuli were presented either haptically (actively palpating an object) or visually (seeing a black-and-white photograph of an object; for a detailed description of the objects, see Object Stimuli), and S1 and S2 were both presented either within the same modality (unimodal) or across modalities (crossmodal). With respect to the event of matching (i.e., processing of S2 and relating it to S1), the experiment resulted in a 2 × 2 × 2 design. The first factor was the mode of sensory matching (unimodal or crossmodal). The second factor related to congruency in object identity between S1 and S2 (congruent or incongruent). The sensory modality of the S2 (visual or haptic) constituted the third experimental factor.

A visual instruction was presented before each stimulus which specified the type of upcoming stimulus (S1 or S2) and whether subjects would see or touch it. An exclamation mark announced an S1, a question mark an S2, a white font a visual stimulus, and a black font a haptic stimulus. The instruction was presented for 0.5 s. A short blank screen of 0.1 s separated instruction and stimulus presentation. S1 and S2 were both presented for 2 s. Inter-stimulus- and inter-trial-intervals (i.e., the time between the offset of an S1 or S2 stimulus, respectively, and onset of the next visual instruction) were randomized between 2 and 6 s in length (in steps of 1 s). During the whole scanning session, the visual display showed a gray background (RGB 128/128/128) on which either the visual objects, the visual instructions, a white fixation cross (inter-stimulus- and inter-trial-interval) or nothing was presented (blank and presentation of haptic objects). Trials were presented pseudo-randomized such that the same objects would not repeat across successive trials. Moreover, the sensory modality combination was repeated maximally once across successive trials. Every object appeared once as S1 and once as S2 in each experimental condition. The combination of S1 and S2 objects in incongruent trials was randomized. Importantly, subjects did not know whether the S2 would be a visual or a haptic object until 0.6 s before its onset (i.e., when a visual instruction informed the subjects about the modality of S2). Thus, all trials with a visual S1 and all trials with a haptic S1 were identical, respectively, until shortly before the onset of S2.

A total of 192 trials (24 trials per condition) were presented during each fMRI experiment. The experiment was split into two runs lasting approximately for 20 min (96 pseudorandomized trials per run). Subjects lay supine in the scanner with their right hand on the right side of a custom-made board fixed by a vacuum-cushion onto their waists. The board was placed such that subjects were comfortably able to reach the placement area in the middle of the board with their forearm and hand without moving either the upper arm or neck muscles. Their left hand was placed beside the body and rested on the button box. Subjects were presented with a white fixation cross and instructed to wait for a visual instruction. When presented with a black sign, they were asked to move their right hand toward the placement area and explore the presented object. During visual and haptic stimulus presentations, the fixation cross disappeared. Subjects were trained to keep a pace of maximally 2 s for hand movements and exploration, and they were asked to repose their hand after the fixation cross reappeared. In case of a white sign, they were asked to look at the following visually presented object until the fixation cross reappeared. At the presentation of S2, subjects were instructed to indicate by button press as fast and accurately as possible whether both objects were the same or different. Responses were made with the middle and index finger of the left hand, with the finger-response assignment being counterbalanced across subjects. Visual stimuli were presented using Presentation (Neurobehavioral Systems, Albany, CA, USA) running on a Windows XP professional SP3 PC. Visual stimuli (objects subtended 8° × 8° and instructions 0.6° × 1.6° on a background of 23° × 12° of visual angle) were back-projected onto a screen using a LCD projector (PROxtraX, Sanyo, Munich, Germany) visible to the subjects through a mirror mounted on the MR head coil. Haptic stimuli were exchanged by the investigator, and the individual objects were always placed in the same viewpoint. The investigator was informed by auditory instructions one trial in advance about which object had to be placed and also about the start and ending of trials. Thus, the investigator was able to control that the haptic stimuli were palpated within the required time frame.

### Object stimuli

The stimulus description is reproduced from (Kassuba et al., 2013a: Stimuli, p. 62) with slight changes in phrasing. Visual and haptic object stimuli were identical for the localizer and the experimental task [same as in Kassuba et al. (2011, 2013a)]. They were manipulable man-made hand-sized objects that the subjects palpated with their right hand. Object categories were restricted to tools, toys, and musical instruments. All objects were real-sized and composed of the same material as in the real world so that they were familiar to the subjects. Furthermore, the objects were deliberately chosen to have an original size such that the objects were easy to palpate and manipulate with one hand. Identical objects appeared in both sensory modalities. Visual object stimuli were black/white photographs taken from the objects used as haptic stimuli. The objects were photographed from the corresponding viewpoint as they were presented to the participants in the haptic condition, and centered on a 350 × 350 pixel sized square consisting of a vertical gray gradient going from RGB 108/108/108 to 148/148/148.

### MRI data acquisition

The study was carried out on a 3 Tesla MRI scanner with a 12-channel head coil (TRIO, Siemens, Erlangen, Germany). We acquired 38 transversal slices (216 mm FOV, 72 × 72 matrix, 3 mm thickness, no spacing) covering the whole brain using a fast gradient echo T2^*^-weighted echo planar imaging (EPI) sequence (TR 2480 ms, TE 30 ms, 80° flip angle). High-resolution T1-weighted anatomical images were additionally acquired after the localizer fMRI scan using an MPRAGE (magnetization-prepared, rapid acquisition gradient echo) sequence (256 mm FOV, 256 × 192 matrix, 240 transversal slices, 1 mm thickness, 50% spacing, TR 2300 ms, TE 2.98 ms).

### Repetitive TMS

Focal rTMS was applied off-line outside the MR scanner room using a figure-of-eight coil attached to a Magstim Rapid stimulator (Magstim Company, Dyfeld, UK). The coil was centered over the left LO using Brainsight frameless stereotaxy (Rogue Research, Montreal, Canada). The center of the eight-shaped coil targeted the MNI coordinates in mm: *x* = −42, *y* = −63, *z* = −3 as determined by the localizer (for details see Epoch-related fMRI Localizer). For each subject, the group peak LO coordinates were transformed into individual anatomical MRI native space coordinates, and the site of rTMS stimulation was verified and traced throughout the conditioning with the frameless stereotaxy device.

Subjects received continuous 1 Hz rTMS for 30 min (1800 stimuli). Stimulation intensity was set to 110% of the individual resting motor threshold (RMT) of the right first dorsal interosseous muscle. Mean stimulation intensity during real rTMS was 53.00 ± 7.50% of total stimulator output. The RMT was defined as the lowest stimulus intensity that evoked a motor evoked potential (MEP) of 50 μV in five out of ten stimuli given over the motor hot spot. Besides the stimulation intensity, the rTMS protocol was identical the protocol used by Siebner et al. (2003) which had resulted in a suppression of neuronal activity in the stimulated left dorsal premotor region that was measurable for at least 1 h after the end of stimulation. In the current study, stimulation intensity was increased to account for the greater scalp-cortex distance of the target region compared to primary motor cortex (Stokes et al., 2005). Repetitive TMS was well tolerated by all participants apart from one female subject who aborted the real rTMS session because of uncomfortable sensations on her skull. Four of the remaining 18 subjects displayed slight twitches in neck and jaw muscles during real rTMS. Repetitive TMS of the left LO did not produce phosphenes in any subject.

MEPs were recorded from the first dorsal interosseous muscle with Ag-AgCl electrodes attached to the skin using a tendon-belly montage. Electromyographic responses were amplified, filtered, and sampled using a D360 eight-channel amplifier (Digitimer, Welwyn Garden City, UK), a CED 1401 analog-to-digital converter (Cambridge Electronics Design, Cambride, UK), and a personal computer running Signal software (Cambridge Electronic Design). The sampling rate was 5 kHz, and signals were band-pass filtered between 5 and 1000 Hz.

An air-cooled figure-of-eight coil (double 70 mm cooled coil system; Magstim Company) was used for real rTMS. The coil was placed tangential to the skull with the handle pointing backward, parallel to the horizontal and the mid-sagittal plane (Ellison and Cowey, 2006). For sham rTMS, a non-charging standard figure-of-eight coil (double 70 mm coil; Magstim Company) was placed at the skull instead, and the charging coil was placed 90° tilted on top of the non-charging coil. In order to provide a comparable acoustic stimulus, intensity of the charging coil was increased for 15% of the total stimulator output. In analogy to the sham rTMS condition, the non-charging coil was placed 90° tilted on top of the charging coil during real rTMS in order to keep the real and sham rTMS conditions as similar as possible.

Repetitive TMS conditioning was performed offline before fMRI but after the short object recognition and task training session. On average, it took 10 ± 2 min from the end of rTMS until fMRI data acquisition was started. This time was needed to move the subjects from the TMS lab to the MR scanner, bed them, set up the board for haptic stimulus presentation, and localize the FOV. Since previous neuroimaging studies have shown that 1Hz rTMS conditioning can produce effects on regional neuronal activity that last for up to 1 h after the end of stimulation (Lee et al., 2003; Siebner et al., 2003), fMRI lasted 40 min and was, thus, within the time limits for capturing reorganizational effects.

### Behavioral data analysis

The description of the behavioral data analysis is reproduced from (Kassuba et al., 2013a: Behavioral analysis, p. 62) and adjusted to include rTMS-specific analysis steps. For each subject and for each trial condition, mean RTs relative to the onset of S2, and response accuracies were calculated. Only correct responses were considered for further analyses (trials excluded due to errors: 0–5 per subject/condition, overall Median = 0; *M* ± *SD* sham rTMS session 0.69 ± 0.84 trials, real rTMS session 0.81 ± 1.07 trials, *p* = 0.32). Haptic trials in which participants did not palpate the object, dropped the object, or made premature or late palpations, as well as palpations lasting longer than 2 s were excluded from analysis (sham rTMS session 0.04 ± 0.08 trials, real rTMS session 0.01 ± 0.03 trials, *p* = 0.41). Within each participant and condition, RTs that differed ±3 standard deviations from the preliminary mean were defined as outliers and excluded from further analyses (sham rTMS session 0.29 ± 0.16 trials, real rTMS session 0.29 ± 0.19 trials). Mean RTs of the adjusted data were entered into a repeated-measures ANOVA (PASW Statistics 18) with rTMS (real/sham), S2-MODALITY (visual/haptic), CONGRUENCY (congruent/incongruent), and SENSORY-MATCHING (unimodal/crossmodal) as within-subject factors. In order to capture transient effects of rTMS conditioning on behavior, RTs within each condition were divided into four time bins of about 10 min each (~4-7 trials/bin). Additional ANOVAs with the factors TIME and rTMS were run for each S1-S2 condition. Each of these ANOVAs tested for a linear trend in the factor Time, and whether this trend interacted with rTMS. Statistical effects at *p* < 0.05 were considered significant. *Post-hoc* Bonferroni corrected paired *t*-tests were used to test for differences between single conditions.

### Functional MRI data analysis

The basic steps of the fMRI analysis is reproduced from Kassuba et al. (2013a: Functional image analysis, pp. 62–63) with slight changes in phrasing and adjustments to include rTMS-specific analysis steps. Image processing and statistical analyses were performed using SPM8 (statistical parametric mapping 8; www.fil.ion.ucl.ac.uk/spm). The first five volumes of each time series were discarded to account for T1 equilibrium effects. Data processing consisted of slice timing (correction for differences in slice acquisition time), realignment (rigid body motion correction) and unwarping (accounting for susceptibility by movement interactions), spatial normalization to MNI standard space as implemented in SPM8, thereby resampling to a voxel size of 3 × 3 × 3 mm^3^, and smoothing with an 8 mm full-width at half-maximum isotropic Gaussian kernel.

Statistical analyses were carried out using a general linear model approach. The time jitter between the onsets of S1 and S2 allowed us to model the effects of rTMS on sample encoding (response to S1) and target matching (response to S2) independently. At the individual level (fixed effects), we defined separate regressors for the onsets of S1 and S2 in each session (i.e., after sham and real rTMS): two different S1 regressors (one for visual S1 and one for haptic S1; Vx and Hx) and eight different S2 regressors (one for each matching condition: V, visual; H, haptic; c, congruent; i, incongruent: VVc, HVc, VVi, HVi, HHc, VHc, HHi, VHi) for each rTMS condition. Only onsets of S1 and S2 in correct trials withstanding the same inclusion criteria as applied for RT analyses were included. An additional regressor modeled the onsets of S1 and S2 in all excluded trials (errors, improper haptic exploration, and outliers) combined over all conditions. All onset vectors were modeled by convolving delta functions with a canonical hemodynamic response function as implemented in SPM8 and their first derivative. Low frequency drifts in the BOLD signal were removed by a high-pass filter with a cut-off period of 128 s. On the group level, we evaluated effects of rTMS on sample encoding (onset S1), target matching (onset S2) as well as time dependent effects.

#### Sample encoding (Onset S1)

In order to determine the modulation of visual (Vx) and haptic (Hx) S1 encoding by rTMS on the group level (random effects), a flexible factorial design with the within-subject factors MODALITY (Vx/Hx) and rTMS (r/s) was configured. The model also included the estimation of the subjects' constants in form of a SUBJECT factor, and accounted for a possible non-sphericity of the error term (dependences and possible unequal variances between conditions in the within-subject factors).

#### Target matching (Onset S2)

Given the complexity of the design (rTMS × S2-MODALITY × CONGRUENCY × SENSORY-MATCHING: 2 × 2 × 2 × 2), we aggregated the S2 matching conditions (S2-MODALITY × CONGRUENCY × SENSORY-MATCHING) into one S2-Condition factor (SPM does not allow a specification of more than 3 factors in a factorial model). In order to evaluate the modulation of S2 processing in a random effects group analysis, we configured a flexible factorial design with the within-subject factors rTMS (r/s) and S2-CONDITION (VVc/HVc/VVi/HVi/HHc/VHc/HHi/VHi). The model also included the estimation of the subjects' constants in form of a SUBJECT factor, and accounted for a possible non-sphericity of the error term (dependences and possible unequal variances between conditions in the within-subject factors). Note that in order to evaluate S2 matching effects, we first calculated contrasts of interest for visual and haptic S2 conditions separately (e.g., crossmodal > unimodal × congruent > incongruent for haptic S2: [VHc - HHc] > [VHi - HHi], for visual S2: [HVc - VVc] > [HVi - VVi]). This enabled us to eliminate modality-specific confounding factors such as residual effects of the cue on S2 processing, eye movements or potential visual imagery and motor activations during haptic but not visual exploration. In a next step, we compared these modality-specific differential effects across modalities (instead of comparing visual and haptic S2 processing directly).

#### Time-dependent effects of rTMS

Time-dependent effects on the processing of S1 and matching of S2 were also investigated in order to capture transient effects of rTMS on task-related neuronal processing which gradually recovered during the ~40 min fMRI session. In each session, each of the two S1 processing conditions (Vx, Hx) was divided into 10 time bins (5 time bins per run) of about 4 min each (~7-10 trials/bin). In the single subject analysis, we defined a regressor for each time bin in each condition. For each condition, we defined contrasts that represented a linear or an exponential modulation over time (i.e., across successive time bins). The exponential function we modeled was *y* = a + (b · 2^−x^), where *y* is the BOLD signal and *x* is time. The beta images of these contrasts of all subjects in the real rTMS and the sham rTMS sessions were then entered into a random effects flexible factorial model [cf. Sample Encoding (Onset S1)] in order to compare time-dependent effects between real and sham rTMS sessions on the group level.

We applied the same approach to the analysis of S2 responses. Here, each S2 matching condition was divided into four time bins of about 10 min each (~4-7 trials/bin) and fitted to a linear function. A division into more than four time bins was not reasonable given the limited number of trials. Given only four time bins for the S2 matching conditions, non-linear time-dependent effects were not modeled here.

#### Regions of interest

The description of the regions of interest is reproduced from Kassuba et al. (2013a: Functional image analysis, p. 63) with slight changes in phrasing and adjustments. We report voxel-wise family wise error rate (FWE) corrected *p*-values as obtained from small volume correction in visuo-haptic regions of interest (ROIs; *p* < 0.05). Four brain regions were predefined as ROIs: LO, FG, aIPS, and pIPS. The ROIs in left and right LO and FG were delineated from the localizer. Images of the localizer data were preprocessed and analyzed as reported previously (Kassuba et al., 2011). Converging object-specific processing across vision and haptics was calculated with a conjunction of the respective object > texture contrasts within each modality. Only voxels that showed an absolute increase during object processing vs. baseline fixation were included. Small volume correction was based on spheres of 8 mm radius centered at the group-based peak coordinates obtained from the conjunction contrast thresholded at *p* < 0.001, uncorrected: *x* = −42, *y* = −63, *z* = −3 for the left LO (rTMS target), *x* = 48, *y* = −69, *z* = −9 for the right LO, *x* = −36, *y* = −39, *z* = −21 for the left FG, and *x* = 36, *y* = −45, *z* = −27 for the right FG.

Four additional ROIs in the left and right aIPS and pIPS were derived from previous studies applying a crossmodal matching task. Correction was based on spheres of 8 mm radius centered at group-based peak coordinates reported by the previous studies. Talairach coordinates (Talairach and Tournoux, 1988) from previous studies were transformed into MNI standard space (mm) as implemented in SPM8 using a MATLAB code provided by BrainMap (http://brainmap.org/icbm2tal/index.html; Lancaster et al., 2007). The spherical ROIs were centered over the stereotactic coordinates *x* = −42, *y* = −40, *z* = 40 for the left aIPS (Grefkes et al., 2002), *x* = −28, *y* = −65, *z* = 49 for the left pIPS (Saito et al., 2003), and *x* = 31, *y* = −62, *z* = 50 for the right pIPS (Saito et al., 2003). We also included the right hemispheric homolog of the left aIPS as a region of interest (*x* = 42, *y* = −40, *z* = 40). Whole-brain voxel-wise FWE correction was applied for all other voxels in the brain (*p* < 0.05). Activations derived from the whole-brain analyses were anatomically labeled using the probabilistic stereotaxic cytoarchitectonic atlas implemented in the SPM Anatomy Toolbox version 1.8 (Eickhoff et al., 2005), adjusted based on anatomical landmarks in the average structural T1-weighted image of all subjects. Percent signal changes used for visualization of the results were extracted using the SPM toolbox rfxplot (Gläscher, 2009).

## Results

### Behavioral performance

**Figure 3:**
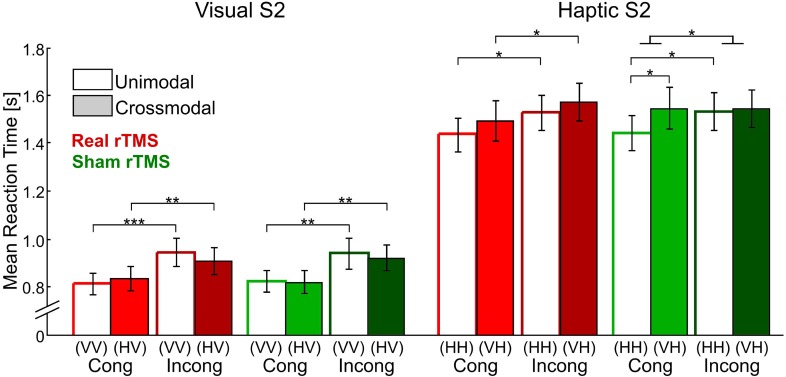
**Mean reaction times for visual and haptic S2 for all four delayed matching conditions (unimodal/crossmodal × congruent/incongruent) and after real (red bars) and sham rTMS (green bars) conditions**. Error bars indicate the standard error of the mean. Reaction times were recorded from S2 onset onwards. Repetitive TMS did not have any effects on reaction times. Sample-target (S1–S2) conditions: V, visual; H, haptic. Cong, congruent; Incong, incongruent. ^*^p < 0.05, ^**^p < 0.01, ^***^p < 0.001, Bonferroni corrected.

Task performance after sham rTMS has been reported in a previous paper (Kassuba et al., 2013a). In short, RTs were longer for incongruent than for congruent trials [*F*_(1,17)_ = 31.43, p < 0.001], indicating that incongruent matching was in general more demanding than congruent matching. RTs decreased linearly during the fMRI session in all conditions [*F*_(1,17)_ = 14.37, *p* < 0.01]. Response accuracies were nearly perfect irrespectively of condition (on average 96.76 ± 0.97% correct). Neither response accuracies nor RTs (time-dependent and time-independent effects) were affected by rTMS conditioning (*p* > 0.10, see Figure 3 and Supplementary Table S1).

### Functional MRI

The fMRI results after sham rTMS have been reported in a previous paper (Kassuba et al., 2013a).

#### Sample encoding (response to S1)

**Figure 4:**
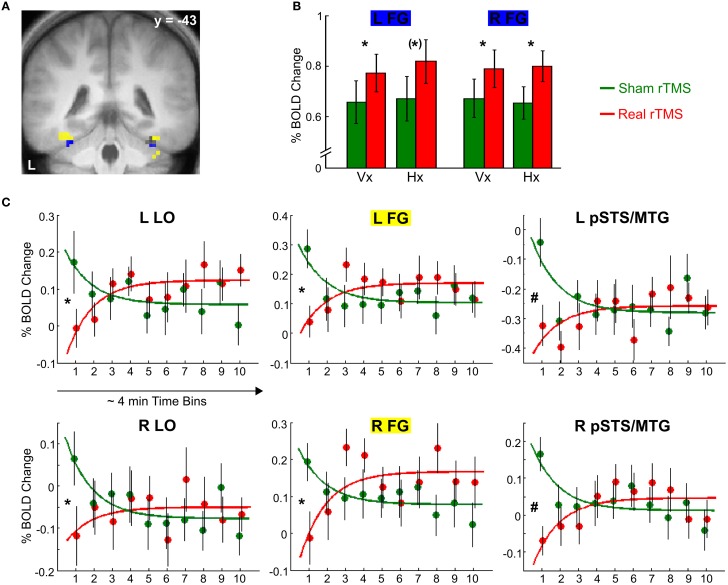
**Effects of real rTMS on S1 processing**. **(A)** Activation map showing temporally stable increases in activation (blue) and transient decreases in activation (yellow) in bilateral FG after real compared to sham rTMS (*p* < 0.01, uncorrected). **(B)** Temporarily stable increases in activation in bilateral FG (blue portion in **(A)**, MNI coordinates *x*, *y*, *z*; left: −33, −46, −23; right: 33, −43, −23) to both visual S1 (Vx) and haptic S1 (Hx) after real (red) relative to sham rTMS (green). **(C)** Transient rTMS-induced decreases in activation during haptic S1 encoding. Regional activity in bilateral LO, FG, and pSTS/MTG showed an interaction of exponential time-dependent effects by rTMS condition when haptic S1 were processed: Whereas regional activity was initially decreased and exponentially increased over time after real rTMS (red), the reversed pattern was found after sham rTMS (green). Similar but weaker effects were found for visual S1 processing (*p* < 0.05, uncorrected). Each time bin represents ~4 min and 7–10 trials. FG, fusiform gyrus [yellow portion in **(A)**, left: −36, −46, −20; right: 36, −43, −20]; LO, lateral occipital cortex (left, i.e., rTMS target area: −42, −67, −11; right: 45, −73, −5); pSTS/MTG, posterior superior temporal sulcus /middle temporal gyrus (left: −66, −40, 1; right: 54, −40, −8). L, left; R, right. ^*^p < 0.05, small volume corrected, (^*^)*p* = 0.052, small volume corrected, ^#^*p* < 0.05, whole brain corrected.

Bilateral LO, FG, aIPS, and pIPS were all activated during visual and haptic S1 encoding both after sham and real rTMS [*t*_(51)_ ≥ 5.30, *p* < 0.001, corrected]. This mean response to S1 was increased in an inferior portion of bilateral FG [left: −33, −46, −23, *t*_(51)_ = 2.86, *p* = 0.052, corrected; right: 33, −43, −23, *t*_(51)_ = 3.46, *p* < 0.05, corrected; see Figures 4A,B] after real as opposed to sham rTMS but otherwise did not differ between the two sessions (*p* > 0.01, uncorrected).

Real TMS affected the activity at the site of stimulation (left LO) mainly during S1 encoding and in a time-dependent fashion. After real rTMS, the BOLD response at the left LO to haptic S1 was initially attenuated and exponentially recovered until ~30 min post rTMS [−42, −67, −11; *t*_(51)_ = 3.49, *p* < 0.05, corrected; see Figure 4C]. The regional BOLD response to haptic S1 stimuli displayed opposite temporal dynamics after sham rTMS with a higher initial level of S1-induced activity which quickly attenuated during continuous task performance. Relative to sham rTMS, real rTMS additionally caused a transient attenuation of haptic S1 processing in the right LO [45, −73, −5; *t*_(51)_ = 3.37], a superior portion of bilateral FG [left: −36, −46, −20, *t*_(51)_ = 3.74; right: 36, −43, −20, *t*_(51)_ = 3.16], and bilateral posterior superior temporal sulcus and adjacent middle temporal gyrus [pSTS/MTG; left: −66, −40, 1; *t*_(51)_ = 6.10; right: 54, −40, −8, *t*_(51)_ = 5.44; all *p* < 0.05, corrected; see Figures 4A,C]. Similar but weaker (*p* < 0.05, uncorrected) transient decreases in activation were found for visual S1 encoding as well. The effects for haptic S1 were not significant different from the effects for visual S1 (*p* > 0.05, corrected).

#### Target matching (response to S2)

**Figure 5:**
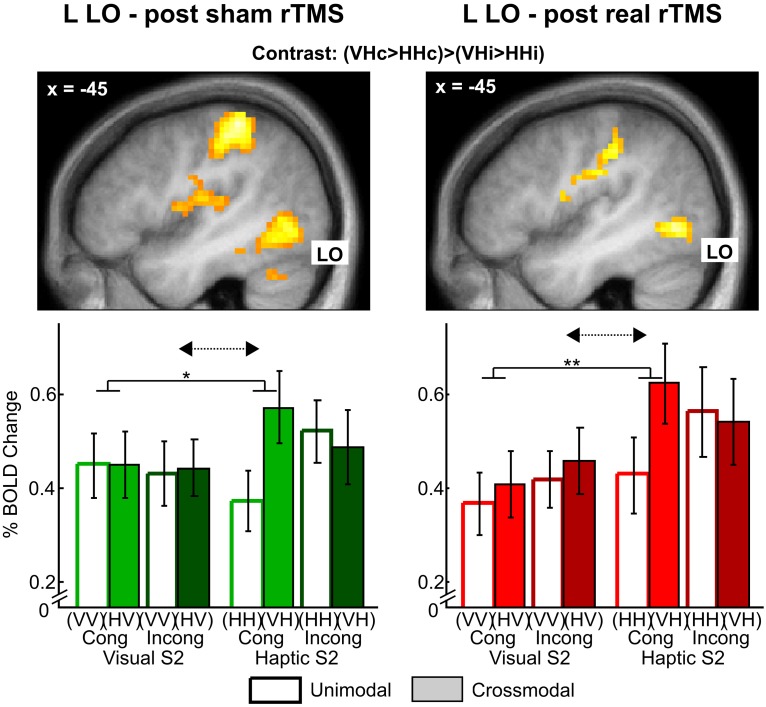
**Stronger multisensory interaction effects for haptic vs. visual S2 in left lateral occipital cortex (LO) after sham (left) and real rTMS (right)**. **Top:** Activation maps showing a crossmodal matching by semantic congruency interaction effect (crossmodal > unimodal × congruent > incongruent) for haptic S2. For illustrative purposes, the statistical maps are thresholded at *p* < 0.01, uncorrected, and overlaid on the average structural T1-weighted image of all subjects. **Bottom:** Percent signal change and error bars indicating the standard error of the mean for each condition at the left LO (MNI coordinates: *x*, *y*, *z* = −45, −70, −5). Stronger visuo-haptic interaction effects for haptic as opposed to visual S2 (solid lines: congruent crossmodal > unimodal × haptic S2 > visual S2, ^*^p < 0.05, ^**^p < 0.01, corrected; dashed arrows: crossmodal > unimodal x congruent > incongruent × haptic S2 > visual S2, *p* ≤ 0.063, corrected) were not affected by rTMS (*p* > 0.01, uncorrected). Sample-target (S1-S2) conditions: V, visual; H, haptic; Cong, congruent; Incong, incongruent. For further significant results see Supplementary Tables S2–S4. The results after sham rTMS have been previously published in Kassuba et al. (2013a).

***Effects of real rTMS on crossmodal congruent matching***. We expected rTMS to evoke the strongest reorganizational effects for crossmodal matching of semantically congruent stimulus pairs (i.e., in the crossmodal matching by semantic congruency interaction contrast as indication for multisensory interactions). After sham rTMS, we had found such multisensory interaction effects in bilateral LO, FG, aIPS, and pIPS which were more pronounced for haptic than visual S2 (Kassuba et al., 2013a). Based on these findings, we proposed that multisensory interactions are more likely for haptic than visual object recognition, and we, therefore, expected stronger effects of real rTMS for the matching of haptic as opposed to visual S2. After real rTMS, we found comparable multisensory interaction effects in our ROIs that were stronger pronounced for haptic as opposed to visual S2 conditions (see Figure 5 and Supplementary Tables S2–S4). We did not observe any significant effects of rTMS on multisensory interactions (rTMS x crossmodal > unimodal × congruent > incongruent) nor on crossmodal matching effects (rTMS × crossmodal > unimodal), neither for visual nor haptic S2.

However, real rTMS altered the temporal dynamics of event-related activity during crossmodal matching compared to sham rTMS. Several regions in left temporal cortex showed initial increases in activations after real rTMS during crossmodal matching of congruent onjects (see Table 1). These effects of real rTMS were transient and decreased gradually during the fMRI session, resulting in a negative linear modulation of the BOLD response. For congruent crossmodal matching of haptic S2 (VHc), the left FG showed an initial relative enhancement of the BOLD response to S2 after real rTMS with a subsequent linear decay over time. In contrast, for congruent crossmodal matching of visual S2 (HVc), the left temporal pole and pSTS/MTG displayed an initial increase in S2-related activation after real rTMS (see Table 1). Direct comparisons between the two modalities (r-VHc > s-VHc × time vs. r-HVc > s-HVc × time) showed that these effects were modality specific. No consistent effects of real rTMS were found during unimodal matching in these regions. Yet, the effects found for crossmodal matching did not differ significantly from the effects for unimodal matching.

**Table 1:** **Linear time-dependent effects of rTMS on regional activity during crossmodal congruent matching**.

**Region**	***x***	***y***	***z***	***t*_peak_**	***p*_corr_(*p*_uncorr_)**	
**HAPTIC S2 (VHc): POST-REAL rTMS > POST-SHAM rTMS × Time**						**>VISUAL S2**
L FG	−42	−34	−20	3.03	0.024^§^	0.066^§^
R anterior parahippocampus	24	−1	−29	3.76	(<0.001)	(<0.001)
R precentral gyrus	39	−7	52	3.95	(<0.001)	0.032
**VISUAL S2 (HVc): POST-REAL rTMS > POST-SHAM rTMS × Time**						**>HAPTIC S2**
L temporal pole	−51	−4	−29	5.85	0.001	0.004
L pSTS/MTG	−66	−40	−2	4.87	0.034	(<0.001)

Coordinates are denoted by x, y, z in mm (MNI space) and indicate the peak voxel. The last column shows the direct comparison of the respective effect to the corresponding effect of the other S2 modality condition. Strength of activation is expressed in t- and p-values corrected for the whole brain and uncorrected p-values in parentheses, respectively, at peak voxel (df = 119),

§ *small volume corrected. FG, fusiform gyrus; pSTS/MTG, posterior superior temporal sulcus/middle temporal gyrus. L, left; R, right*.

***Effects of real rTMS on incongruent matching***. Longer response latencies suggested that matching of incongruent objects was behaviorally more challenging than matching of congruent objects (see Figure 3). Since behavioral performance was not impaired by rTMS, we next asked whether we could find reorganizational effects on the neuronal level related to incongruent matching, that is, triggered by task difficulty. We found rTMS-induced increases in activations related to matching of incongruent objects for both haptic and visual S2. These effects were found transiently for crossmodal matching of haptic S2 and lastingly (i.e., temporally stable for the whole duration if the experiment) for unimodal matching of visual S2 (see Table 2). When a haptic S2 was matched to an incongruent visual S1 (r-VHi > s-VHi), real rTMS-induced transient increases in activation were found in bilateral parahippocampus, right LO, bilateral pSTS/MTG, IPS, and in the right middle and adjacent superior frontal gyrus. On the other hand, when a visual S2 was matched to an incongruent visual S1, temporarily stable increases in activation were found in the left FG and pIPS. No other incongruent matching condition was affected by real rTMS.

**Table 2:** **Linear time-dependent effects of rTMS on regional activity during crossmodal incongruent matching**.

**Region**	***x***	***y***	***z***	***t*_peak_**	***p*_corr_(*p*_uncorr_)**	
**TEMPORALLY STABLE EFFECTS: POST-REAL rTMS > POST-SHAM rTMS**						
**Haptic S2—crossmodal: VHi**						
No significant results						
**Haptic S2—unimodal: HHi**						
No significant results						
**Visual S2—crossmodal: HVi**						
No significant results						
**Visual S2—unimodal: VVi**						**>haptic S2**
L FG	−33	−43	−26	2.70	0.051^§^	0.289^§^
L pIPS	−30	−64	55	2.91	0.034^§^	0.192^§^
**LINEAR TIME DEPENDENT EFFECTS: POST-REAL rTMS > POST-SHAM rTMS**						
**Haptic S2—crossmodal: VHi × time > visual S2**						**>visual S2**
L parahippocampus / FG	−36	−16	−20	5.69	0.001	(<0.001)
R middle frontal gyrus	39	−4	61	5.46	0.004	0.014
R superior frontal gyrus	27	5	64	5.13	0.013	(<0.001)
R anterior parahippocampus	24	−1	−26	5.28	0.007	
L pSTS/MTG	−48	−73	13	5.14	0.013	(<0.001)
R pSTS/MTG	42	−70	10	4.66	(<0.001)	(<0.001)
R LO	48	−73	−2	3.56	0.006^§^	0.024^§^
L aIPS	−36	−37	37	3.55	0.006^§^	0.002^§^
L pIPS	−21	−67	49	3.65	0.005^§^	0.001^§^
R pIPS	27	−61	46	2.99	0.026^§^	0.017^§^
**Haptic S2—unimodal: HHi × time**						
No significant results						
**Visual S2—crossmodal: VHi × time**						
No significant results						
**Visual S2—unimodal: VVi × time**						
No significant results						

Coordinates are denoted by x, y, z in mm (MNI space) and indicate the peak voxel. The last column shows the direct comparison of the respective effect to the corresponding effect of the other S2 modality condition. Strength of activation is expressed in t- and p-values corrected for the whole brain and uncorrected p-values in parentheses, respectively, at peak voxel (df = 255 for temporally stable effects, df = 119 for time-dependent effects),

§ *small volume corrected. aIPS, anterior intraparietal sulcus; FG, fusiform gyrus; LO, lateral occipital cortex; pIPS, posterior IPS; pSTS/MTG, posterior superior temporal sulcus/middle temporal gyrus. L, left; R, right*.

**Figure 6:**
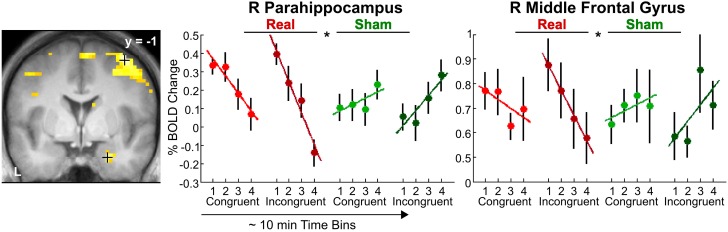
**Transient incongruency effects for crossmodal matching of haptic S2 (VH) evoked by rTMS conditioning**. Regional activity in the right parahippocampus (MNI coordinates: *x*, *y*, *z* = 24, −1, 29) and right middle frontal gyrus (39, −1, 58) showed an interaction of linear time-dependent effects by rTMS condition that was stronger for incongruent than congruent trials: Whereas regional activity was initially increased and linearly decreased over time after real rTMS (red, real-congruent; dark red, real-incongruent), no significant linear time-dependent increases in activations (or rather decreases) were found after sham rTMS (green, sham-congruent; dark green, sham-incongruent), and these differential effects were stronger for incongruent than congruent conditions (real > sham × incongruent > congruent × time). For illustrative purposes, the statistical maps are thresholded at *p* < 0.001, uncorrected, and overlaid on the average structural T1-weighted image of all subjects. Each time bin represents ~10 min and 4–7 trials. L, left; R, right. ^*^p < 0.05, corrected.

**Figure 7:**
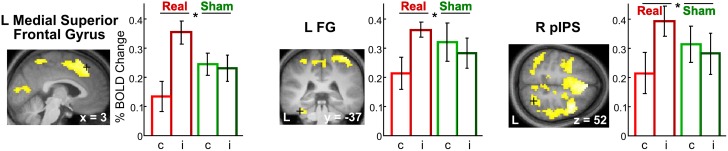
**Incongruency effects for unimodal visual matching (VV) evoked by rTMS conditioning**. Only after real but not sham rTMS, regional activity in the L superior medial gyrus (MNI coordinates: *x*, *y*, *z* = 3, 32, 52) **(left)**, L FG (−36, −37, −26) **(middle)**, and R pIPS (27, −58, 52) **(right)** was increased when a visual S2 was matched to an incongruent (i) as compared to a congruent (c) visual S1 (real > sham × incongruent > congruent). The same effects were found in the left pIPS. For illustrative purposes, the statistical maps are thresholded at *p* < 0.001, uncorrected, and overlaid on the average structural T1-weighted image of all subjects. Bars represent percent signal change and error bars the standard error of the mean for each VV condition after real and sham rTMS (red, real-congruent; dark red, real-incongruent; green, sham-congruent; dark green, sham-incongruent). FG, fusiform gyrus; pIPS, posterior intraparietal sulcus; L, left; R, right. ^*^p < 0.05, corrected.

***Incongruency effects (incongruent > congruent) after real rTMS***. The time-dependent effects in the right anterior parahippocampus and middle frontal gyrus and adjacent precentral gyrus found for crossmodal matching of haptic S2 were significantly more pronounced for incongruent than congruent conditions (real > sham × VHi > VHc × time, see Table 3 and Figure 6). Thus in these regions, real rTMS conditioning induced incongruency effects, that is, stronger activations during incongruent than congruent matching, that were not evident after sham rTMS. Such rTMS by incongruency interactions (real > sham × incongruent > congruent) were found for unimodal visual (VV) matching as well. For unimodal visual matching, temporarily stable rTMS-induced incongruency effects were found the left superior medial gyrus extending to the right hemisphere, left FG, and bilateral pIPS (see Table 3 and Figure 7). A direct comparison of visual and haptic S2 conditions showed that these time-dependent ([r-VHi > s-VHi × time] > [r-HVi > s-HVi × time]) and time-independent effects ([r-VVi > s-VVi] > [r-HHi > s-HHi]) were modality-specific. Unimodal matching of haptic S2 and crossmodal matching of visual S2 did not show real rTMS-induced incongruency effects.

**Table 3:** **Real rTMS induced incongruency effects (incongruent > congruent × real rTMS > sham rTMS)**.

**Region**	***x***	***y***	***z***	***t*_peak_**	***p*_corr_(*p*_uncorr_)**	
**TEMPORALLY STABLE EFFECTS: POST-REAL rTMS > POST-SHAM rTMS**						
**Haptic S2—crossmodal: VHi > VHc**						
No significant results						
**Haptic S2—unimodal: HHi > HHc**						
No significant results						
**Visual S2—crossmodal: HVi > VVc**						
No significant results						
**Visual S2—unimodal: VVi > VVc**						**>haptic S2**
L superior medial gyrus	3	32	52	4.92	0.014	(<0.001)
L FG	−36	−37	−26	4.55	<0.001^§^	0.039^§^
L pIPS	−30	−61	55	3.81	0.002^§^	0.092^§^
R pIPS	27	−58	52	3.73	0.003^§^	0.050^§^
**LINEAR TIME DEPENDENT EFFECTS: POST-REAL rTMS > POST-SHAM rTMS**						
**Haptic S2—crossmodal: VHi > VHc × time > visual S2**						**>visual S2**
R anterior parahippocampus	24	−1	−29	5.60	0.002	(<0.001)
R middle frontal gyrus	39	−1	58	5.61	0.002	0.004
**Haptic S2—unimodal: HHi > HHc × time**						
No significant results						
**Visual S2—crossmodal: VHi > VHc × time**						
No significant results						
**Visual S2—unimodal: VVi > VVc × time**						
No significant results						

Coordinates are denoted by x, y, z in mm (MNI space) and indicate the peak voxel. The last column shows the direct comparison of the respective effect to the corresponding effect of the other S2 modality condition. Strength of activation is expressed in t- and p-values corrected for the whole brain and uncorrected p-values in parentheses, respectively, at peak voxel (df = 255 for temporally stable effects, df = 119 for time-dependent effects),

§ *small volume corrected. FG, fusiform gyrus, pIPS, posterior intraparietal sulcus. L, left; R, right*.

#### Exclusion of subjects with low LO activations in the localizer

One concern with respect to the null findings regarding multisensory interactions could be that we used the peak coordinates from the localizer group analysis as rTMS target instead of individual peaks. Yet theoretically, the group peak coordinates represent the peak responses across subjects, and indeed, the Eucledian distance between individual peaks and the group peak were smaller than 1 cm in all subjects. However, 5 out of the 18 subjects showed very weak activations in the localizer contrast and peaks in the left LO could only be localized at very low thresholds (*p* > 0.05, uncorrected). In these subjects, the group peak coordinates provided a more objective guide for placing the TMS coil. To test whether these subjects had biased our results, we repeated our analyses without these 5 subjects. There were still no significant effects of rTMS on multisensory interactions.

## Discussion

We probed short-term plasticity of visuo-haptic object recognition by conditioning neuronal processing in left LO with low-frequency offline rTMS. Compared to sham rTMS, real rTMS led to a dynamic redistribution of brain activity during visuo-haptic object matching. Changes in task-related activity were not only triggered in the stimulated left and contralateral LO but also in remote temporal and parietal regions previously associated with object recognition. While LO, FG, aIPS, pIPS have been implicated in visuo-haptic object recognition (Amedi et al., 2001; Grefkes et al., 2002; Saito et al., 2003; Kassuba et al., 2011), the pSTS/MTG seems to participate in audio-visual and audio-haptic object recognition (Beauchamp et al., 2004, 2008; Kassuba et al., 2011, 2013b), and the temporal pole appears to support semantic memory (Martin and Chao, 2001; Rogers et al., 2006). Since behavioral performance was not impaired, the real rTMS-induced changes in task-related brain activity likely indicate compensatory processes preserving behavior after neuronal challenge. Importantly, the pattern of real rTMS-induced changes in regional activity differed as a function of the stage of the delayed-match-to-sample task (S1 encoding vs. S2 matching) and the target modality.

Since various previous studies have implicated the left LO in visuo-haptic integration of object information (Lacey and Sathian, 2011), we predicted that rTMS of the left LO would particularly affect multisensory interactions as defined by crossmodal matching by semantic congruency interactions and particularly for haptic S2 conditions (Kassuba et al., 2013a,b). Contrary to our expectations, rTMS had no impact on crossmodal matching effects (crossmodal > unimodal) regardless of whether or not semantic congruency was considered and neither for visual nor haptic S2.

### Attenuated response to S1 but not S2 at the site of stimulation (left LO)

However, in accordance with a suppressive effect on regional neuronal activity (Gerschlager et al., 2001; Siebner et al., 2003) focal 1 Hz rTMS of the left LO temporarily decreased the neural response to S1 in the stimulated region. This decrease in activity was primarily observed during haptic S1 processing in left LO with only a weak trend of deactivation for visual S1. The suppressive effect of rTMS on haptic processing involved the whole LOC and pSTS/MTG bilaterally, indicating a spread of the suppressive effect of rTMS to other posterior cortical areas presumably via cortico-cortical connections. Together, the findings show that rTMS to the left LO selectively suppressed haptic processing of S1 but not S2 in the stimulated LO. This context-dependent effect on haptic processing suggests that 1 Hz rTMS primarily suppressed regional neural activity in the left LO related to more explorative haptic processing (S1) without affecting a more comparative processing (S2) of objects in a delayed match-to-sample context.

Using the current design (Kassuba et al., 2013a) or an analogous design with auditory and haptic stimuli (Kassuba et al., 2013b), we have previously reported a dissociation between S1 and S2 processing related to an adaptation of the BOLD response due to the repeated presentation of objects with the same identity over the duration of the experiment. Only S1 encoding but not S2 matching showed reduced responses as a function of how often an object had been already presented throughout the experiment. We speculate that S1 encoding and S2 matching represent distinct functional states, the former might be more bottom-up driven while the latter might be more top-down dependent. As a consequence, left LO conditioning leads to different reorganizational changes.

### Increased responses to S2 in remote regions

While processing of S2 was unchanged at the site of stimulation, transient increases in activation emerged in remote regions after real relative to sham rTMS in congruent crossmodal matching trials, that is, when object concepts were most likely integrated across the senses (Laurienti et al., 2004). These putatively compensatory increases in activation were found in temporal regions such as the left temporal pole and pSTS/MTG for crossmodal matching of visual S2 (HVc) and the left FG and right anterior parahippocampus for crossmodal matching of haptic S2 (VHc) and were specific for the respective S2 modality. It has been previously proposed that the temporal cortex integrates object information (e.g., object motion, shape, use-associated motor movements) with increasing convergence and abstraction along the posterior to anterior axis (Martin and Chao, 2001; Martin, 2007). For instance, studies that used dynamic visual and auditory object stimuli suggested that the pSTS/MTG is tuned to features of motion associated with different objects (Beauchamp et al., 2002, 2004). We have previously shown that the same left FG region as found here shows object-specific responses independent of whether objects were seen, heard, or touched, suggesting more abstract or conceptual representations of object information (Kassuba et al., 2011, 2013b; see also Martin, 2007). Patient studies suggest that the anterior temporal pole is critical for semantic memory (Rogers et al., 2006) and particularly for retrieving object information about unique entities (Damasio, 1989; Damasio et al., 1996). We, therefore, propose that in the presence of a functional perturbation of the left LO, regions of a semantic object recognition network are increasingly activated when the same objects are matched across vision and haptics. These enhanced activations might reflect a compensatory strategy involving semantic memory. Critically, retrieving haptic object information and matching it to the same object processed visually activated different nodes of this putative network than retrieving visual object information and matching it to the same object processed haptically.

One likely explanation for the null findings with respect to real rTMS effects on multisensory interactions is that the delayed-match-to-sample task was not challenging enough. Even after real rTMS, task accuracy was nearly perfect (≥95%). We found real rTMS-induced increases in activations in LOC and IPS related to matching of incongruent objects, which was behaviorally more difficult than matching of congruent objects. Some of these increased brain activations were specifically stronger during incongruent than congruent matching (incongruency effect), only after real but not sham rTMS. Again, these rTMS-induced incongruency effects differed based on the S2 modality: The effects were limited to the first 30 min post rTMS in the right anterior parahippocampus for crossmodal haptic matching of haptic S2 (VH) but remained stable throughout the session in left FG and bilateral pIPS for unimodal matching of visual S2 (VV). Even though left LO rTMS had no effects on multisensory interactions, these results suggest a functional relevance of left LO for evaluating visual and haptic object information.

Since S1 and S2 were presented sequentially, incongruency effects (incongruent > congruent) could also be interpreted as repetition suppression or fMRI-adaptation (fMRI-A) effects (i.e., decreased activity in the congruent condition due to the repeated presentation of objects with the same identity). Thus, incongruency effects found for crossmodal matching could be interpreted as crossmodal adaptation and might indicate multisensory integration (cf. Tal and Amedi, 2009; Doehrmann et al., 2010; Van Atteveldt et al., 2010). However, we argue that the task demands in our paradigm have overruled general effects of stimulus habituation (for a detailed discussion of this issue, see Kassuba et al., 2013a,b). First, the stimulus onset asynchronies between S1 and S2 in the present study were rather long and favored a semantic encoding of S1. Second, while other studies showing adaptation effects typically used a task orthogonal to the effect of interest such as a detection task (Doehrmann et al., 2010; Van Atteveldt et al., 2010; Snow et al., 2013) or passive recognition (Tal and Amedi, 2009), our task required an explicit semantic decision on the identity of S1 and S2. In addition, using this delayed-match-to-sample paradigm, we did not find any general adaptation of the BOLD response to S2 due to repeated presentations of the same objects throughout the experiment (independent of matching condition), neither when using visual and haptic stimuli (Kassuba et al., 2013a), nor when using auditory and haptic stimuli (Kassuba et al., 2013b). Consistent with our findings, other studies employing longer delays in visuo-haptic priming (James et al., 2002) or using a delayed-match-to-sample task (Grefkes et al., 2002) have found enhanced instead of decreased BOLD responses in LO and IPS to crossmodal matching. Thus, the transient rTMS-induced incongruency effects for crossmodal matching of haptic S2 most likely reflect an increased response to incongruent stimuli after real rTMS. We speculate that this increase is due to compensatory activations that help to maintain task performance in the behaviorally most challenging condition.

### Methodological considerations

The null effects of rTMS with respect to behavioral performance and multisensory interactions have to be interpreted in light of the stimulus paradigm and applied rTMS stimulation protocol. In addition to semantic congruency, temporal and spatial coherence are important factors for multisensory integration (Stein and Stanford, 2008). We presented crossmodal stimuli sequentially (instead of simultaneously) and in different positions with respect to the subjects' egocentric spaces (visual: mirror on head coil, haptic: on the subjects waist). The delayed-match-to-sample task enabled us to identify a differential contribution of vision and haptics to visuo-haptic interactions and guaranteed that objects were processed conceptually. Therefore, our paradigm rather probed visuo-haptic interactions in higher-order object recognition than basic visuo-haptic integration. In addition, behavioral performance was at ceiling. Thus, the delayed-match-to-sample task might have not been sensitive enough to identify rTMS effects on multisensory interactions, behaviorally or neurally.

Previous studies in which LO TMS had been found to impair visual object processing have used different tasks and applied TMS “online” (i.e., while participants performed the task). For example, Ellison and Cowey (2006) used discrimination tasks with simultaneously presented shapes and applied a high-frequency five-pulse train at stimulus onset. In the study by Pitcher et al. (2009), subjects performed a delayed-match-to-sample task as well, although with shorter presentation times (500ms S1 + 500 ms mask + 500 ms S2) than in the present study and TMS was applied to the right LO. In that study, the online administration of a 10 Hz TMS train was aligned with S2. These studies have applied TMS online during the task and not as a conditioning offline protocol as we did in the present study.

It is important to recall that the effects of online and offline rTMS are not the same (Siebner and Rothwell, 2003; Siebner et al., 2009b). With its prolonged effects on cortical excitability, offline rTMS induces a complex reorganization and re-weighting of the involvement of cortical structures in task relevant networks (Siebner et al., 2009a). The system may adapt to the rTMS-induced changes to maintain functional homeostasis. Effects of rTMS conditioning on behavior are typically reported in the first 15 min post rTMS (cf. Rounis et al., 2006; O'Shea et al., 2007; Mancini et al., 2011), while effects on neuronal activity can be measured up to 1 h post rTMS (Siebner et al., 2003). Our fMRI measurement started on average 10 min post rTMS. There are previous studies that found changes in neuronal activity at the stimulated region and in remote regions after 1 Hz rTMS conditioning without affecting behavior later than 10 min post rTMS (Lee et al., 2003; O'Shea et al., 2007). Therefore, the lack of behavioral impairment but task-related changes in cortical activity found in the current study could be interpreted as functional reorganization preserving behavior after neuronal challenge.

It is possible that we would have found behavioral effects if fMRI had started earlier within the first 10 min post rTMS. The rTMS-related effects might have been stronger if we had used individual activations from the localizer as rTMS target regions instead of the peak response from the group analysis. However, individual peak responses were close to the group peak. Further, results did not change when we excluded 5 subjects from the analyses that showed only weak visuo-haptic convergence in the left LO during the localizer.

## Conclusions

The fact that we found distinct effects for different S2 matching conditions supports the idea that these reflect compensatory mechanisms provoked by task demands rather than mere transsynaptic spreading of rTMS conditioning. Together, the results support the notion that the left LO is functionally relevant for both visual and haptic object recognition but to a different extent. Our data suggest that visuo-haptic object recognition involves a network of regions comprising the bilateral LO, FG, aIPS, pIPS, pSTS/MTG, and anterior temporal regions, which can be flexibly recruited if the system is challenged. How compensatory processing is allocated depends on the target modality (visual vs. haptic) and task demands (S1 encoding vs. S2 matching).

## Author contributions

Tanja Kassuba and Hartwig R. Siebner conceived the experiment. Tanja Kassuba, Cordula Hölig, and Hartwig R. Siebner designed the experiment. Tanja Kassuba and Corinna Klinge collected the data. Tanja Kassuba analyzed the data. All authors interpreted the results and wrote the paper.

### Conflict of interest statement

The authors declare that the research was conducted in the absence of any commercial or financial relationships that could be construed as a potential conflict of interest.
