# Supplementary material for: Short-term plasticity of visuo-haptic object recognition
Source: Front Psychol. 2014 Apr 2;5:274. doi: 10.3389/fpsyg.2014.00274 (PMC3980106; doi:10.3389/fpsyg.2014.00274)
Supplement: Supplementary file 1 [file DataSheet1.PDF]

Supplementary Results

Supplementary Table S1. Response Accuracy (Mean±Standard Error of the Mean in Percent) for Each Condition (N = 18)

	**Real rTMS**		**Sham rTMS**	
	**Congruent**	**Incongruent**	**Congruent**	**Incongruent**
**Visual Targets**				
Unimodal (visual-visual)	95.83±1.12	96.99±1.05	96.99±1.00	98.15±0.84
Crossmodal (haptic-visual)	96.74±0.80	97.45±1.02	95.83±0.89	97.22±0.67
**Haptic Targets**				
Unimodal (haptic-haptic)	97.69±0.77	94.91±1.45	95.80±0.68	97.69±0.69
Crossmodal (visual-haptic)	95.60±1.52	97.45±0.96	95.83±1.21	97.92±0.77

Supplementary Table S2. Crossmodal Matching Effects (Crossmodal > Unimodal) for Congruent Object Pairs in Regions of Interest After Sham and Real rTMS

	After-Sham					After-Real		Real vs. Sham
**Region**	***x***	***y***	***z***	t_{peak}	p_{corr}	t_{peak}	p_{corr}	p_{corr}
**Visual S2: HVc > VVc**								
L lateral occipital cortex	-48	-64	-8	2.49	0.098	4.82	**<0.001**	n.s.
R lateral occipital cortex	45	-64	-11	1.91	0.262	3.66	**0.005**	n.s.
L fusiform gyrus	-30	-43	-20	1.25	0.508	3.74	**0.004**	0.075
R fusiform gyrus	33	−46	−26	2.78	**0.041**	3.56	**0.007**	n.s.
L anterior intraparietal sulcus					n.s.		n.s.	
R anterior intraparietal sulcus					n.s.		n.s.	
L post. intraparietal sulcus					n.s.		n.s.	
R post. intraparietal sulcus					n.s.		n.s.	
**Haptic S2: VHc > HHc**								
L lateral occipital cortex	−48	−67	−8	6.12	**<0.001**	7.07	**<0.001**	n.s.
R lateral occipital cortex	54	−64	−8	4.33	**<0.001**	4.80	**<0.001**	n.s.
L fusiform gyrus	−39	−43	−20	5.51	**<0.001**	4.74	**<0.001**	n.s.
R fusiform gyrus	39	−43	−20	3.10	**0.019**	3.06	**0.026**	n.s.
L anterior intraparietal sulcus	−42	−34	46	4.27	**0.001**	4.29	**0.001**	n.s.
R anterior intraparietal sulcus	42	−31	40	3.87	**0.002**	3.81	**0.003**	n.s.
L post. intraparietal sulcus	−24	−70	46	4.76	**<0.001**	4.75	**<0.001**	n.s.
R post. intraparietal sulcus	27	−67	52	2.45	0.079	2.89	**0.042**	n.s.

Coordinates are denoted by *x*, *y*, *z* in mm (MNI space) and indicate the peak voxel after sham rTMS. Strength of activation is expressed in *t*-scores and small volume corrected *p*-values at the peak voxel ($df = 119$ for the results after sham/real rTMS, $df = 255$ for the comparison of real vs. sham rTMS). Post. = posterior, L = left, R = right. Sample−target (S1−S2) conditions: V = visual, H = haptic, c = congruent. n.s. = not significant ($p > 0.01$, uncorrected). The results after sham rTMS have been previously published in Kassuba et al. (2013), Neuroimage, 65, Table 1, p. 64.

Supplementary Table S3. Crossmodal Matching by Congruency Interaction (Crossmodal > Unimodal x Congruent > Incongruent) After Sham and Real rTMS

	After-Sham					After-Real		Real vs. Sham
**Region**	***x***	***y***	***z***	t_{peak}	p_{corr}	t_{peak}	p_{corr}	p_{corr}
**Visual S2: (HVc > VVc) > (HVi > VVi)**								
L lateral occipital cortex	-51	-70	-8	1.81	0.306	3.58	**0.006**	n.s.
R lateral occipital cortex	51	-64	-11	1.38	0.463	2.56	0.083	n.s.
L fusiform gyrus	-39	-46	-20	0.72	0.663	2.64	0.071	n.s.
R fusiform gyrus	36	-43	-20	2.31	0.137	2.76	0.054	n.s.
**Haptic S2: (VHc > HHc) > (VHi > HHi)**								
L lateral occipital cortex	−42	−70	−8	3.78	**0.003**	4.17	**0.001**	n.s.
R lateral occipital cortex	42	−67	−5	2.67	0.052	3.08	**0.025**	n.s.
L fusiform gyrus	−42	−34	−20	3.23	**0.014**	2.70	0.061	n.s.
R fusiform gyrus	39	-46	-29	2.54	0.089	2.91	**0.038**	n.s.
L anterior intraparietal sulcus	−42	−40	49	4.14	**0.001**	3.74	**0.004**	n.s.
R anterior intraparietal sulcus	39	−34	43	2.82	**0.038**	2.15	0.195	n.s.
L post. intraparietal sulcus	−24	−70	46	3.12	**0.018**	2.72	0.062	n.s.
R post. intraparietal sulcus	36	−64	52	2.99	**0.024**	2.29	0.152	n.s.

Coordinates are denoted by *x*, *y*, *z* in mm (MNI space) and indicate the peak voxel after sham rTMS. Strength of activation is expressed in *t*-scores and small volume corrected *p*-values at the peak voxel ($df = 119$ for the results after sham/real rTMS, $df = 255$ for the comparison of real vs. sham rTMS). Post. = posterior, L = left, R = right. Sample−target (S1−S2) conditions: V = visual, H = haptic, c = congruent, i = incongruent. n.s. = not significant ($p > 0.01$, uncorrected). The results after sham rTMS have been previously published in Kassuba et al. (2013), Neuroimage, 65, Table 2, p. 64.

Supplementary Table S4. Stronger Visuo-Haptic Interaction Effects for Haptic Versus Visual Targets in Regions of Interest

**Region**	***x***	***y***	***z***	t_{peak}	$p_{corrected}$					
					**(1) S**	**(1) R**	**(1) R vs. S**	**(2) S**	**(2) R**	**(2) R vs. S**
L LO	−45	−70	−5	3.07	**0.020**	**0.001**	n.s.	0.057	0.063	n.s.
R LO	51	−64	−5	2.93	**0.029**	**0.049**	n.s.	0.083	0.621	n.s.
L FG	−39	−43	−20	3.85	**0.002**	**0.049**	n.s.	0.077	0.205	n.s.
L aIPS	−42	−40	49	2.73	**0.045**	**0.002**	n.s.	**0.023**	0.106	n.s.
R aIPS	39	−34	43	3.24	**0.013**	**0.002**	n.s.	0.263	0.319	n.s.

Coordinates are denoted by *x*, *y*, *z* in mm (MNI space) and indicate the peak voxel in the two-way interaction contrast (1) after sham rTMS (S). Strength of activation is expressed in *t*-scores at the peak voxel (*df* = 119 for the results after sham/real rTMS, *df* = 255 for the comparison of real vs. sham rTMS). Small volume corrected *p*-values are indicated for the following contrasts (from left to right): (1) crossmodal-congruent > unimodal-congruent x haptic > visual: (VHc > HHc) > (HVc > VVc); (2) crossmodal > unimodal x congruent > incongruent x haptic > visual: [(VHc > HHc) > (VHi > HHi)] > [(HVc > VVc) > (HVi > VVi)]), S = after sham rTMS, R = after real rTMS. aIPS = anterior intraparietal sulcus, FG = fusiform gyrus, LO = lateral occipital cortex. L = left, R = right. Sample−target (S1−S2) conditions: V = visual, H = haptic, c = congruent, i = incongruent. n.s. = not significant ($p > 0.01$, uncorrected). The results after sham rTMS have been previously published in Kassuba et al. (2013), Neuroimage, 65, Table 3 p. 65.
